# Lessons from the first two years of operating a study registry

**DOI:** 10.3389/fpsyg.2015.00173

**Published:** 2015-02-18

**Authors:** Caroline Watt, James E. Kennedy

**Affiliations:** ^1^Department of Psychology, University of EdinburghEdinburgh, UK; ^2^Biometrics Department, Allos Therapeutics, Inc.Westminster, CO, USA (retired)

**Keywords:** study registration, study registry, confirmatory research, confirmation, research methodology

The need for improved methodology for psychological research has recently received much attention. The primary recommendation has been increased emphasis on confirmatory or replication research that is carefully planned with adequate sample size and is pre-registered (Wagenmakers et al., 2012; Nosek and Lakens, 2014; Simons et al., 2014). Study registration options are currently being developed and implemented. Based on our experience operating a study registry, we offer practical recommendations and observations that may be useful when implementing study registration more widely.

In the fall of 2012, we opened a study registry at the University of Edinburgh's Koestler Parapsychology Unit (KPU) (KPU Registry, 2012). Consistent with the standards for registering clinical trials (International Committee of Medical Journal Editors, 2005), the registry focuses on public, prospective registration with specified registration information, and is not affiliated with a specific journal. The present discussion addresses methodology, not the findings of the registered studies. Parapsychological researchers have strived to utilize the established research methods of experimental psychology. This aspiration has resulted in increasing publications in high profile psychology journals (Bösch et al., 2006; Storm et al., 2010; Bem, 2011), but has not provided noticeable progress in resolving the debates about parapsychology. This situation was a significant factor in the recognition by psychologists that improved research methodology was needed (Pashler and Wagenmakers, 2012; Wagenmakers et al., 2012).

Based on experience working in regulated medical research, the second author has long advocated that the standard research methods for academic psychology were not adequate for controversial research like parapsychology and that formal, pre-registered, well-powered confirmatory research was needed (Kennedy, 2004). The first author also pointed out the value of pre-registered confirmatory research (Watt, 2005). However, these proposals received little interest at that time. The limitations of the common psychological research methods became increasingly apparent over the years and we began developing the KPU Registry (2012). As we were starting to send notices that the registry was open, a group of articles was published (Pashler and Wagenmakers, 2012) that significantly increased awareness of the need for these practices. Discussions of study registration now usually focus on how registration should be done rather than whether registration is beneficial. In the present paper we make several recommendations for avoiding pitfalls and obtaining the full benefits of study registration.

## Exploratory and confirmatory research

Distinguishing between exploratory and confirmatory research is important for study registration. Registration has high value for confirmatory research, but less value for exploratory research. Exploratory research is typically the creative step that is the starting point for a line of research, whereas confirmatory research provides the convincing evidence that makes science valid and self-correcting. This distinction is usually straightforward for regulated medical research—Phase 1 and Phase 2 studies are exploratory, and Phase 3 studies are confirmatory (National Library of Medicine, 2008). However, the social sciences have not had these clear distinctions and labels (De Groot, 1956/2014; Wagenmakers et al., 2012). Studies in the social sciences often have both exploratory and confirmatory components.

We recommend that each registered hypothesis or analysis be classified as exploratory or confirmatory. For a study pursuing only exploratory analyses, registration in the KPU registry is considered optional. The strongest evidence pertaining to an effect comes from registered confirmatory research. Meta-analysis of exploratory research does not eliminate the need for well-designed confirmatory research (Cooper and Hedges, 2009; Ferguson and Heene, 2012). A guidance document was developed to help experimenters distinguish between exploratory and confirmatory analyses (KPU Registry, 2014b).

Confirmatory research has two key characteristics. First, confirmatory research can provide evidence that the hypothesis of interest is false as well as true. This implies that the study has adequate sample size and that the measurement methods and experimental interventions are established. Studies are exploratory if they involve the development of measurement methods or new experimental interventions that could confound the interpretation of evidence for or against the primary hypothesis. Also, non-significant results for an underpowered study are ambiguous because the results could be due to low power rather than to the experimental hypothesis being false.

Second, all analysis decisions that could affect the confirmatory results are made prior to the start of data collection. These decisions include the specific statistical methods, the criteria for acceptable evidence, any transformations or adjustments to the data, and any criteria for excluding or deleting data.

## Specifying the analysis

We recommend that the analysis decisions noted above be included in the registration information for confirmatory hypotheses. This amount of detail about the planned analysis is greater than typically required to register a clinical trial, but is less extensive than the statistical analysis plan that regulatory agencies expect for confirmatory studies (International Conference on Harmonisation, 1998). Experimenters submitting to the KPU registry frequently omitted required information from their initial registration information. A document with checklists and examples for classical, Bayesian, and classification analyses was recently developed to assist experimenters in providing the needed information (KPU Registry, 2014a). For exploratory analyses, less detail is acceptable for registration, and it is recognized that the analysis methods may need to be developed or modified as the data are being analyzed.

## The need for review

We recommend that the submitted registration information is reviewed to verify that the required information has been provided. Reviews of the KPU registrations found deficiencies for virtually all initial submissions. Common omissions included not specifying whether analyses were one or two sided and not specifying the prior probability distributions for Bayesian analyses.

Ambiguities and inconsistencies about the independent and dependent variables also occurred. In one case the planned hypothesis test was presented as confirmatory and the type of statistical test was pre-specified as an ANOVA. However, the scores from a questionnaire were the independent variable for the ANOVA, and the experimenter did not specify the criteria for mapping the scores to discrete categories for the analysis. For a confirmatory analysis, the criteria for assigning the categories needed to be pre-specified in order to document that the experimenter did not explore different criteria during data analysis and select the criteria that produced the most favorable results. This specification was requested as part of the registration review, and the ambiguity was eliminated.

We now believe that impartial, detailed review of the completeness and consistency of registration information is essential. We have found that authors of registry submissions welcome the review process and recognize that it strengthens study registration and enhances the credibility of the study. The primary clinical trials registry, ClinicalTrials.gov (2010), also requires certain registration information and reviews submitted information for completeness and consistency.

## Additional benefits of registration

Registries maintain information about studies indefinitely and are pivotal for literature reviews. For medical research, study registries are often the starting point for reviews. Widely used and easily searched registries allow reviewers to find efficiently the strongest evidence and unpublished studies. In addition, registries increasingly provide links or abstracts for the study results. The KPU registry encourages experimenters to provide links or information about the experimental results that can be subsequently posted with the registration information.

In addition to preventing common research biases, public study registration promotes scientific efficiency and reduces unintended duplication of research effort (International Committee of Medical Journal Editors, 2005). Registration can also serve a social function of letting others know about the research activities for a researcher or for an institution.

## What registration does not do

Basic study registration publicly documents the key planned methodology for a study, but does not evaluate whether the methodology is adequate. For example, the registration process does not consider whether other statistical methods would be preferable, whether the planned experimental procedures preclude alternative explanations, or whether the planned hypotheses are meaningful. These types of questions are most effectively handled with peer review prior to registration of the final study plan. Peer review of the planned methodology can be obtained by privately circulating a description of the study among colleagues or by posting the description on the internet and inviting comments prior to formal registration on a public registry. Journals that will accept a study based on peer review of the planned methodology enhance study quality and are increasingly available (Chambers, 2014; Simons et al., 2014; Taylor and Francis Group, 2014; Chambers et al., 2015). However, these *registered reports* do not replace all the benefits of public, prospective, searchable registration. Easily searched public registration can be a required step for registered reports.

Study registration also does not prevent fraud by an experimenter. Other methodological practices are needed to prevent fraud and are appropriate for confirmatory research (Stroebe et al., 2012; Kennedy, 2014).

## Irreversibly public prospective registrations

The KPU registry and the major medical registries have substantially simpler, faster registration processes and more flexible publication options than the registered replication reports for specific journals, but also have substantially greater structure than the self-registration process of Open Science Framework (OSF). OSF provides online processes for managing scientific documents, data, and collaboration, and includes an option for registration (OSF, 2011-2014). OSF is probably the best-known registration option for psychologists.

Registration at OSF consists of making a copy of the electronic study documents and assigning a date-time stamp to the copy. A registration copy cannot be changed or deleted, but the experimenter controls the content of a registration, how many registrations are made, and whether a registration is kept private or made public. This process allows an experimenter to examine the study results before deciding whether to make the study and/or registration public (OSF, 2011-2014), and to reset a public registration back to private (verified functionally on OSF and by OSF support in March, 2014). The associated *Preregistered badge* does not require that a registration was irreversibly public before data collection started (Blohowiak et al., 2014). As of December, 2014, OSF does not provide registrations that are irreversibly public.

For comparison, the standards for clinical trials and for the KPU registry are that registrations are controlled by an independent organization that has certain minimum registration requirements and makes the registrations irreversibly public before data collection starts. This eliminates the options to keep or make registrations private if the results are unfavorable. If the experimenter can examine the study results before deciding whether to make a registration public, experiments with favorable outcomes can be presented as pre-registered, but experiments with unfavorable outcomes may be kept privately in the file drawer as the experimenter moves on to other higher priorities. This substantially compromises the value of study registration. A relatively simple registration process with greater structure and improved search capabilities could be implemented within OSF or could be a feature of new registries.

## Summary of recommendations

Our recommendations for study registration are concisely listed below. This list identifies key factors for registration and may be useful for those planning to register a study or managing a study registry. The recommendations are:

- public registration before data collection has begun;
- registrations cannot be removed or made private after data collection has started;
- each hypothesis or analysis classified as exploratory or confirmatory;
- methodology for confirmatory research specified in sufficient detail to document that all decisions that affect the outcome were made prior to any knowledge of the study data;
- registration information independently reviewed for completeness and clarity;
- history of changes publicly displayed for any revisions to the registration information after data collection has begun;
- registration information openly and freely available to anyone (no website login or membership required);
- registrations easily and reliably searched to find all registered studies on a particular topic or by a particular researcher—for literature reviews and future verification of original study plans;
- formal or informal peer review of the planned study prior to registration;
- abstracts or links provided for final study results.

### Conflict of interest statement

The authors declare that the research was conducted in the absence of any commercial or financial relationships that could be construed as a potential conflict of interest.
